# High glucose upregulates BACE1-mediated Aβ production through ROS-dependent HIF-1α and LXRα/ABCA1-regulated lipid raft reorganization in SK-N-MC cells

**DOI:** 10.1038/srep36746

**Published:** 2016-11-10

**Authors:** Hyun Jik Lee, Jung Min Ryu, Young Hyun Jung, Sei-Jung Lee, Jeong Yeon Kim, Sang Hun Lee, In Koo Hwang, Je Kyung Seong, Ho Jae Han

**Affiliations:** 1 Department of Veterinary Physiology, College of Veterinary Medicine, Research Institute for Veterinary Science and BK21 PLUS Program for Creative Veterinary Science Research Center, Seoul National University, Seoul 08826, Korea; 2 Department of Veterinary Physiology, College of Veterinary Medicine, Chonnam National University, Gwangju 61186, Korea; 3 Medical Science Research Institute, Soonchunhyang University Seoul Hospital, Seoul, Republic of Korea; 4 Department of Biochemistry, Soonchunhyang University College of Medicine, Cheonan, 330-930, Republic of Korea; 5 BK21 PLUS Program for Creative Veterinary Science Research, and Research Institute for Veterinary Science; Seoul National University and Korea Mouse Phenotyping Center (KMPC), Seoul, Korea; 6 Department of Anatomy and Cell Biology; Korea Mouse Phenotyping Center (KMPC); College of Veterinary Medicine; Seoul National University, Seoul, Korea

## Abstract

There is an accumulation of evidence indicating that the risk of Alzheimer’s disease is associated with diabetes mellitus, an indicator of high glucose concentrations in blood plasma. This study investigated the effect of high glucose on BACE1 expression and amyloidogenesis *in vivo*, and we present details of the mechanism associated with those effects. Our results, using ZLC and ZDF rat models, showed that ZDF rats have high levels of amyloid-beta (Aβ), phosphorylated tau, BACE1, and APP-C99. *In vitro* result with mouse hippocampal neuron and SK-N-MC, high glucose stimulated Aβ secretion and apoptosis in a dose-dependent manner. In addition, high glucose increased BACE1 and APP-C99 expressions, which were reversed by a reactive oxygen species (ROS) scavenger. Indeed, high glucose increased intracellular ROS levels and HIF-1α expression, associated with regulation of BACE1 and Liver X Receptor α (LXRα). In addition, high glucose induced ATP-binding cassette transporter A1 (ABCA1) down-regulation, was associated with LXR-induced lipid raft reorganization and BACE1 localization on the lipid raft. Furthermore, silencing of BACE1 expression was shown to regulate Aβ secretion and apoptosis of SK-N-MC. In conclusion, high glucose upregulates BACE1 expression and activity through HIF-1α and LXRα/ABCA1-regulated lipid raft reorganization, leading to Aβ production and apoptosis of SK-N-MC.

Several epidemiological and biological evidence sources support a link between diabetes mellitus (DM) and Alzheimer’s disease (AD)^1^,^2^,^3^,^4^. In addition, there is evidence that shows a link between alteration of glucose metabolism and the accumulation of amyloid precursors in brain of diabetic patients^5^,^6^. Although the molecular and pathophysiological mechanisms triggering the occurrence of AD are still not fully described, some studies have suggested that the accumulation and deposition of amyloid-beta (Aβ), which results from inadequate processing of amyloid precursor protein (APP), may contribute to the pathogenesis of AD^7^,^8^. Although several studies suggest DM may be a cofactor for AD occurrence, its presence is insufficient to produce AD occurrence^9^,^10^; however, recent studies have reported that a high glucose environment can aggravate AD pathogenesis via APP accumulation, Aβ production, and plaque formation^11^,^12^,^13^. These findings suggest that investigation into the role of glucose in Aβ production and APP processing is required for developing strategies for the prevention of AD occurrence and treatment of AD in patients who have high blood glucose profile. Beta-site APP cleaving enzyme 1 (BACE1) is a key APP processing enzyme associated with membrane bound C-terminal fragment C99 (APP-C99) and Aβ production. Several studies have reported that BACE1 regulation is involved in AD pathogenesis including Aβ deposition and Aβ-associated memory impairment^14^,^15^,^16^. Moreover, BACE1 inhibitors have been considered as a potent therapeutic candidate for AD treatment^17^. However, there are few reports describing the effect of glucose on BACE1 expression. Chen RF *et al*. reported that deprivation of oxygen and glucose induces BACE1 accumulation^18^. A detailed description of the mechanism by which high glucose regulates BACE1 could provide a novel clue for controlling AD occurrence in DM patients.

Several reports have suggested that DM and hyperglycemia-altered cholesterol metabolism may play a critical role in neurologic and metabolic dysfunctions^19^,^20^. The liver X receptors (LXRs) including LXRα and LXRβ are key transcription factors that stringently regulate intracellular cholesterol levels via regulation of transcriptional activity of genes involved in cholesterol transport and efflux. More recently, it has been reported that membrane lipid composition has an important role in AD pathogenesis^21^; wherein, local increases in membrane cholesterol affect APP processing, resulting in stimulation of Aβ production^22^. Cholesterol is a critical component of the lipid raft, a specialized membrane microdomain that is involved in protein trafficking, signal transduction, neurotransmission, and ligand-receptor interaction^23^. Furthermore, it has been reported that BACE1 is located in the lipid raft, and a change in lipid raft cholesterol could affect BACE1 activity and result in changes to Aβ production, suggested that lipid raft modification by hyperglycemia may be a potential trigger of AD pathogenesis^24^. However, the relationships between hyperglycemia-induced intracellular cholesterol accumulation and Aβ production, and the related signaling pathways, remain unclear.

The ZDF rat, derived from mutation of the leptin gene in the ZF strain, has a high level of blood glucose and is widely used as a model for studying human DM and its complications^25^. In addition, mouse hippocampal neuron and SK-N-MC cells have been widely used as an *in vitro* neuronal cell model to investigate neuronal pathogenesis of AD^26^,^27^,^28^,^29^. Elucidation of the critical molecules affecting the occurrence of AD under diabetic conditions is important for developing a comprehension of AD pathogenesis and can be helpful in developing novel strategies for treatment and prevention of AD. In the present study, we investigated the effect of high glucose on BACE1 expression and related mechanisms by using *in vivo* and *in vitro*, Zucker Diabetic Fatty (ZDF) rats and SK-N-MC human neuroblastoma cells, respectively.

## Materials and Methods

### Materials

Cells from the SK-N-MC human neuroblastoma, MEF mouse embryonic fibroblast and CACO-2 human colon carcinoma were provided by the Korean Cell Line Bank (Seoul, Korea). Fetal bovine serum (FBS) and serum replacement (SR) for cultivation were purchased from Hyclone (Logan, UT, USA) and Gibco (Grand Island, NY, USA), respectively. The p-Tau (Ser^396^), Tau, presenilin-1, β-actin, lamin A/C, JNK, p-JNK (Thr^183^/Tyr^185^), β-tubulin, caveolin-1, flotillin-2, p-GSK3β (Ser^9^), GSK3β and caspase-9 antibodies were purchased from Santa Cruz Biotechnology (Dallas, TX, USA). The Aβ, BACE-1, APP, HIF-1α, LXR, retinoid X receptor (RXR), and ATP-binding cassette transporter A1 (ABCA1) were obtained from Abcam (Cambridge, MA, USA). Caspase-3 antibody was purchased from Cell Signaling Technology (Beverly, MA, USA). Horse radish peroxidase (HRP)-conjugated rabbit anti-mouse and goat anti-rabbit secondary antibodies were purchased from Thermo Fisher (Waltham, MA, USA). The specificity of Aβ antibody was validated by using gamma secretase inhibitor L-685,458 (Sup Fig. 3). N-acetylcysteine (NAC), D-glucose, L-glucose, PD98059, SP600125, TO901317, methyl-β-cyclodextrin (MβCD), filipin III, fluorescein isothiocyanate (FITC)-conjugated cholera toxin subunit B (CTB), and propidium iodide (PI) were obtained from Sigma Aldrich (St. Louis, MO, USA). Small interfering RNAs (siRNAs) for *bace-1*, and non-targeting were purchased from Dharmacon (Lafayette, CO, USA). *hif-1α* siRNA was purchased from GenePharma (GenePharma, Shanghai, China). Alexa fluor 488- and 568-conjugated secondary antibodies were acquired from Life Technologies (Gaithersburg, MD, USA). All reagents used in this study were of the highest quality commercially available forms.

### Cells

The SK-N-MC, MEF and CACO-2 cells were cultured with 10% FBS, 1% antibiotic-antimycotic solution containing penicillin, streptomycin, and fungizone, and high glucose Dulbeco’s essential medium (DMEM; Gibco). The cells were grown on 6-well plates or in 60 mm dishes in an incubator maintained at 37 °C with 5% CO_2_. Cells were incubated for 72 h and then washed with phosphate buffered solution (PBS). Subsequently, the medium was changed to low glucose DMEM-supplemented culture medium with 1% SR and 1% antibiotic-antimycotic solution. After synchronization for 24 h, cells were washed twice with PBS and placed in SR-supplemented low glucose DMEM with reagents.

### Experimental animals

Male and female heterozygous type (*Lepr*^fa/+^) ZDF rats were acquired from Genetic Models (Genetic Models Inc., Indianapolis, IN, USA) and mated to each other to obtain homozygous ZDF lean control (ZLC) and ZDF rats. Male homozygous ZDF and ZLC rats were used in this study. Rats were housed in a standard animal facility and maintained at a room temperature range of 20–25 °C and 60% humidity with a 12 h light/dark cycle. Purina 5008 diet (Purina Korea, Seoul, Korea), as recommended by Genetic Models, was provided to all rats. All procedures involving rats followed the National Institutes of Health Guidelines for the Humane Treatment of Animals; moreover, the protocols were approved by the Institutional Animal Care and Use Committee of Seoul National University (SNU-140219–1). Rats were euthanized at 16 weeks of age. Brain tissues were extracted and fixed with 4% paraformaldehyde in 0.1 M PBS for 24 h. Fixed brain tissues were stored following infusion with 90% sucrose for cryoprotection and then embedded in O.C.T compound (Sakura Finetek, Torrance, CA, USA). Tissue samples were cryosectioned coronally at 10 μm thick using a cryostat (CM1520; Leica, Wetzlar, Germany) and underwent western blotting and immunohistochemical (IHC) staining.

### Mouse primary hippocampal neuron culture

Mouse hippocampus was isolated from 17 days mouse embryos brain. After dissociation of hippocampus tissue with 0.05% trypsin solution, 2 × 10^5^ hippocampal cells were plated onto poly D-lysine coated 35 mm dish, and neurobasal plating media (Neurobasal media containing B27 supplement [1 ml/50 ml], 0.5 mM glutamine, 25 μM glutamate, 1 mM HEPES, 10% horse serum) is added to dish. Cells were incubated for 24 h. Neuronal cells were cultured with neurobasal feeding media (Neurobasal media containing B27 supplement [1 ml/50 ml], 0.5 mM glutamine, 1 mM HEPES, 10% horse serum) for 12 days. All procedure involving primary hippocampal culture followed the National Institutes of Health Guidelines for the Humane Treatment of Animals; moreover, the protocols were approved by the Institutional Animal Care and Use Committee of Seoul National University (SNU-151116-1).

### Western blot analysis

Post-treatment, cells were collected by using a cell-scraper. Cells were washed twice with cold PBS prior to lysis with RIPA buffer (Thermo Fisher) and a proteinase and phosphatase inhibitor cocktail (Thermo Fisher). After incubation in lysis buffer for 30 min on ice, lysates were centrifugated for 30 min at 15,000 rpm at 4 °C, and the supernatant was collected. Determination of protein concentration in samples was performed by using a bicinchoninic acid (BCA) assay kit (Bio-Rad, Hercules, CA, USA). Protein samples (10 μg) were loaded in 8–15% sodium dodecyl sulfate-polyacrylamide gel (SDS-PAGE) for electrophoresis and transferred to a polyvinylidene fluoride (PVDF) membrane. After protein transfer, the protein-transferred membrane was washed with tris-buffered saline containing 0.1% Tween-20 (TBST) solution {150 mM NaCl, 10 mM Tris-HCl (pH 7.6), and 0.1% Tween-20}. Next, the membrane was blocked with 5% skim milk (Gibco) and 5% bovine serum albumin (Thermo Fisher) in TBST solution. The membrane was then washed three times every 10 min and incubated with primary antibody (1:1,000 dilution) overnight at 4 °C. After incubation with primary antibody, the membrane was washed and sequentially incubated with horseradish peroxidase (HRP)-conjugated secondary antibody (1:10,000 dilution) for 4 h at 4 °C. The western blot bands were detected by using an enhanced chemiluminescence solution (Bio-Rad). Densitometric analysis for quantification was performed by using ImageJ software (developed by Wayne Rasband, National Institutes of Health, Bethesda, MD, USA; http://rsb.info.nih.go.kr/ij/). Full-length gels and blots of key data are included in the supplementary information (Sup. Figs 1 and 2),

### Extraction of mRNA and reverse transcription-polymerase chain reaction

The SK-N-MC cells were extracted by using a commercial RNA extraction kit (TaKaRa Biomedicals, Otsu, Japan). Extracted RNA (1 μg) was reverse-transcripted with a reverse transcription premix kit (iNtRON Biotechnology, Sungnam, Korea) to produce cDNA. The cDNA was amplified by using forward and reverse primers of *lxr-α*, *lxr-β*, and *β-actin*.

### Real-time polymerase chain reaction

The mRNA expression levels of the *lxr-α*, *lxr-β*, *abca1*, *abcg1*, and *β-actin* genes were measured by using a Rotor-Gene 6000 real-time thermal cycling system (Corbett Research, Mortlake, NSW, Australia) with a Quanti NOVA SYBR Green PCR Kit (Qiagen, Hilden, Germany) along with cDNA (1 μg) and mRNA primers. The mRNA primer sequences used in this study are described in Supplemental Table 1. The identity and specificity of the polymerase chain reaction (PCR) products were confirmed by performing melting curve analysis. Normalization of gene expression levels was performed by using the *β-actin* gene as a control.

### Immunohistochemical staining

Fixed brain tissue samples were deparaffinized with xylene and various concentrations of ethanol (100, 90, 70, and 50%). For inactivation of endogenous peroxidase, deparaffinized tissues were incubated with 3% hydrogen peroxide in methanol for 10 min, and then washed with PBS twice. Next, antigen retrieval of tissue samples was performed by incubating tissues with pre-warmed (100 °C) citrate buffer {100 mM, 0.05% Tween 20, pH 6.0 in distilled water (DW)} for 20 min. Subsequently, samples were washed and permeabilized by incubation with 0.5% triton X100 in PBS. Samples were then blocked with 5% normal goat serum (Sigma Aldrich) in PBS for 30 min. Tissue slides were incubated with primary antibodies (1:100 dilution) overnight at 4 °C. After washing three times with PBS, tissue slides were incubated with Alexa fluor 488 and conjugated secondary antibodies (1:100 dilution) and PI for 2 h at 4 °C. Immunostained samples were visualized by using confocal microscopy (Fluoview 3000; Olympus, Tokyo, Japan). All scale bars are 200 μm. The magnifications of images are ×100 and ×200.

### Intracellular reactive oxygen species measurement

Detection of intracellular reactive oxygen species (ROS) was performed by using CM-H_2_DCF-DA staining (DCF-DA, Life Technologies). Cells were detached with 0.25% trypsin and 0.5 mM EDTA, then counted by using a Petroff-Hausser counting chamber. Subsequently, 5 × 10^5^ cells were obtained and suspended in 10 μM DCF-DA in PBS and incubated at 25 °C for 1 h in the dark. After incubation, cells were washed twice with PBS and loaded into a 96-well black plate. Measurement of intracellular ROS was performed by using a luminometer (Victor3, Perkin-Elmer, Waltham, MA, USA).

### Small interfering RNA transfection

The *hif-1α* specific siRNA or non-targeting siRNA as a negative control were transfected to SK-N-MC cells for 24 h with Turbofect transfection reagent (Thermo Fisher) prior to treatment according to the manufacturer’s manual. The concentration of transfected siRNAs was 20 nM. All siRNA sequences used in this study are described in Supplemental Table 2.

### Chromatin immunoprecipitation

Chromatin immunoprecipitation (CHIP) assay was performed by using an EZ-ChIP-Chromatin Immunoprecipitation Kit (EMD Millipore, Billerica, MA, USA) according the manufacturer’s instructions. Samples containing protein-chromatin complexes were incubated with antibodies specific for HIF-1α, IgG and PolIII overnight at 4 °C. Normal IgG and PolIII were used as negative and positive controls, respectively. Immunoprecipitated complexes were eluted with elution buffer (1% SDS, 50 mM Tris-HCl, pH 7.5, and 10 mM EDTA). Eluates were incubated with 5 M NaCl for 4 h at 65 °C, and then incubated with RNase A for 30 min at 37 °C. Next, samples were incubated with 0.5 M EDTA, 1 M Tris-HCl, and proteinase K for 2 h at 45 °C. Sample DNA was extracted by supplied column and amplified by PCR using a designed primer (Supplemental Table 3). One percent of the sample chromatin extract was used as an input. Quantification of binding of protein to the promoter was performed by using real-time PCR.

### Sucrose density gradient fractionation

After treatment, cells were washed with cold PBS and scraped into 1 ml of lysis buffer (500 mM, Na_2_CO_3_, 10 mM EDTA, pH 11 in DW). The proteinase and phosphatase inhibitor cocktail was added to the lysates. Subsequently, lysates were homogenized by using a sonicator (Branson Sonicator 250, Branson Ultrasonic Corp., Danbur, CT, USA) and incubated for 30 min on ice. After protein quantification by performing a BCA assay, the same amount of protein was adjusted to form 2 ml of 45% sucrose in MES-buffered solution [MBS: 25 mM MES (pH6.5), 0.15 M NaCl] and placed in an ultracentrifuge tube (Beckman Coulter, Fullerton, CA, USA). To form a 5–35% discontinuous sucrose gradient, 4 ml of 35% sucrose and 4 ml of 5% sucrose containing MBS were sequentially added to the ultracentrifuge tube. Sample-containing ultracentrifuge tubes were centrifugated at 40,000 rpm for 20 h in an SW41 rotor (Beckman Coulter). Fractionized samples were collected and analyzed by performing western blotting.

### Nuclear fraction preparation

Collected cells were suspended in nuclear fraction lysis buffer {137 mM NaCl, 8.1 mM Na_2_HPO_4_, 2.7 mM KCl, 1.5 mM KH_2_PO_4_, 2.5 mM EDTA, 1 mM dithiothreitol, 0.1 mM PMSF, and 10 mg/ml leupeptin (pH 7.5)}. Cells were suspended by pipetting and incubated for 10 min on ice. Lysates were centrifugated at 3,000 rpm for 5 min at 4 °C. To obtain the non-nuclear fraction sample, lysate supernatant was collected. The residual nuclear pellet was lysed with RIPA lysis buffer for 20 min on ice, and then centrifugated at 15,000 rpm for 30 min at 4 °C. Sample supernatant, comprising the nuclear faction, was collected.

### Immunocytochemistry

Cells placed on a confocal dish (Thermo Fisher) were fixed by incubating with 80% acetone in PBS for 10 min, and then washed three times with PBS. Next, cells were blocked with 5% FBS in PBS to inhibit non-specific binding of proteins. The blocked cells were incubated with primary antibody (1:100 dilution) followed by washing with PBS and then incubated with Alexa fluor secondary antibody, FITC-conjugated CTB, and PI for 2 h at 4 °C. Images were obtained by using a Fluoview 300 confocal microscopy (Olympus). Fluorescence intensity of protein was analyzed by using Image J software (developed by Wayne Rasband, National Institute of Health, Bethesda, MD, USA; http://rsb.info.nih.gov/ij/). We calculate the corrected total cell fluorescence (CTCF, Image J arbitary unit). The equation used to determine CTCF is: CTCF = Integrated density − (Area of selected cell × Mean fluorescence of back ground readings).

### Trypan blue exclusion cell viability assay

After treatment, cell-conditioned medium was collected. Cells were washed with cold PBS and then incubated and suspended with a 0.05% trypsin (Gibco) and 0.5 mM EDTA solution (Sigma Aldrich). Suspended cells were then added to the collected medium along with the cell suspension solution. Soybean trypsin inhibitor (0.05 mg/ml) was added to the cell suspension solution to quench the trypsin activity. Samples were centrifugated at 3000 rpm for 5 min at 4 °C. Subsequently, the supernatant was removed and the residual cell pellet suspended with PBS and trypan blue (0.4%, Sigma Aldrich) to stain the dead cells. Stained and unstained cells were counted by using a Petroff-Hausser counting chamber (Hausser Scientific, Horsham, PA, USA). The equation used to determine cell viability was Cell viability = [{1 − (number of trypan blue-stained cells/number of total cells)} ×100].

### Annexin V/PI fluorescence-activated cell sorter analysis

Annexin V and PI staining to evaluate apoptosis of SK-N-MC cells was performed by using an Annexin V/PI apoptosis detection kit (BD Bioscience, Franklin Lakes, NJ, USA) according to the manufacturer’s manual. Briefly, SK-N-MC culture medium was collected after cell treatment. The SK-N-MC cells were detached and suspended with 0.25% trypsin and a 0.5 mM EDTA solution. Next, the collected medium was added to the cell suspension. Suspended cells were counted by using a Petroff-Hausser counting chamber (Hausser Scientific). Cells (1 × 10^5^) were then resuspended in binding buffer supplied with the apoptosis detection kit and immune-stained with 5 μl of FITC-Annexin V and 5 μl of PI for 15 min in the dark at room temperature. Apoptosis analysis was performed by using flow cytometry (Beckman Coulter). Cell (3 × 10^5^) that had similar levels of side scatter value and cell volume were measured and analyzed by using CXP software (Beckman Coulter).

### Total cholesterol measurement

The total cellular cholesterol level was determined by using a commercial cholesterol measurement kit (BioVision, Mountain View, CA, USA) according to the manufacturer’s instructions. Briefly, 1 × 10^6^ cells were collected, lysed with a mixture of chloroform, isopropanol, and NP-40 to extract cellular lipids. The extracted cellular lipids were dried, and the dried pellet suspended in the cholesterol buffer supplied with cholesterol measurement kit. Next, the enzyme mix supplied with the cholesterol measurement kit was added to the samples, which were then incubated for 1 h at 37 °C. The colorimetric protocol for detection was adopted and measurements were made at 570 nm by using a microplate reader (Bio-Rad).

### Filipin III staining for cholesterol measurement

Cells were fixed with 3% fresh paraformaldehyde for 1 h at room temperature. Cells were washed two times with PBS, and incubate with 1 ml of 1.5 mg glycine in PBS solution for 10 min at room temperature to quench the paraformaldehyde. Cells were incubate with filipin III (Sigma Aldrich) working solution (0.05 mg/ml in PBS/10% FBS) for 2 h at room temperature. Confocal image was acquired by using confocal microscopy (340–380 nm excitation).

### Immunoprecipitation

To detect secreted Aβ in the medium, post-treatment SK-N-MC-conditioned medium was collected. The proteinase and phosphatase inhibitor cocktail (Thermo Fisher) was added to the medium sample. To produce the Aβ-specific antibody conjugated with agarose bead, we performed immobilization by using a commercial co-immunoprecipitation kit (Thermo Fisher) according to the manufacturer’s instructions. Medium samples were incubated with a primary antibody specific for Aβ-conjugated agarose beads for 6 h at 4 °C. Agarose beads were spun-down by centrifugation at 1000 rpm for 1 min and then collected. Collected beads were washed six times with wash buffer. Protein was acquired by incubation in elution buffer supplied with the co-immunoprecipitation kit. Protein in the medium was determined by using a BCA protein quantification assay. The same amount of protein was loaded in 12% SDS-PAGE for electrophoresis and transferred to a PVDF membrane. Protein-transferred membranes were incubated with the Aβ-specific antibody. After incubation with HRP-conjugated secondary antibody, blots were detected by using an ECL solution (Bio-Rad).

### Statistical analysis

All experimental data are presented as a mean ± standard error. Differences among groups were tested by using ANOVA. Comparisons of treatment groups with control groups were performed by applying Bonferroni Dunn tests. A test result with a *p* value < 0.05 was considered significant.

## Results

### Effect of high glucose on Aβ secretion and neuronal cell death *in vivo* and *in vitro*

**Figure 1:**
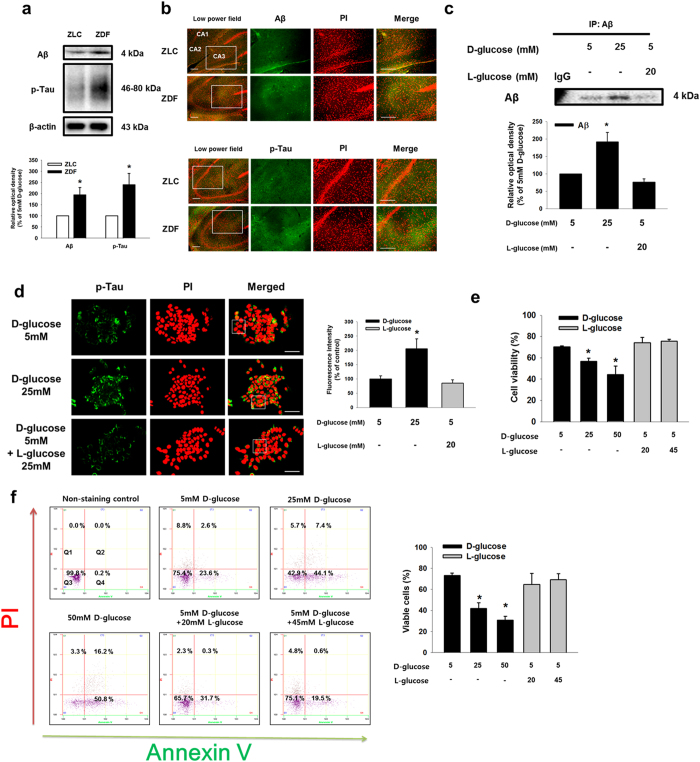
Effects of high glucose on Aβ secretion and neuronal apoptosis. (**a**) Aβ, phosphorylated tau and β-actin protein expressions of the ZLC and ZDF brain tissues were detected by western blotting. (**b**) Slide samples for immunohistochemistry were immunostained with Aβ and p-tau (Ser^396^) specific antibodies and PI. Images shown in result are representative. All scale bars, 200 μm (magnification of low and high power field, ×100 and ×200). (**c**) Secreted Aβ from SK-N-MC in medium was detected by immunoprecipitation assay. Immunoprecipitated Aβ was analyzed by western blotting. (**d**) Cells were immunostained with p-tau (Ser^396^) and PI. Scale bars, 50 μm (magnification, ×800). Fluorescence intensity of p-tau was analyzed by using Image J software (developed by Wayne Rasband, National Institute of Health, Bethesda, MD, USA; http://rsb.info.nih.gov/ij/). The result images are representative of five independent experiments. Data are presented as a mean ± S.E. of six independent experiments. *p < 0.05 versus 5 mM of D-glucose treatment. (**e**) Cell viability was measured by trypan blue exclusion assay Data are presented as a mean ± S.E. of three independent experiments with duplex dishes. (**f**) Viable cells were detected by using annexin V/PI analysis. Data are presented as a mean ± S.E. of two independent duplex dishes. Each western blot result shown is representative images of three independent experiments. *p < 0.05 versus 5 mM of D-glucose treatment. All western blot data were cropped and acquired under same experimental conditions.

**Figure 2:**
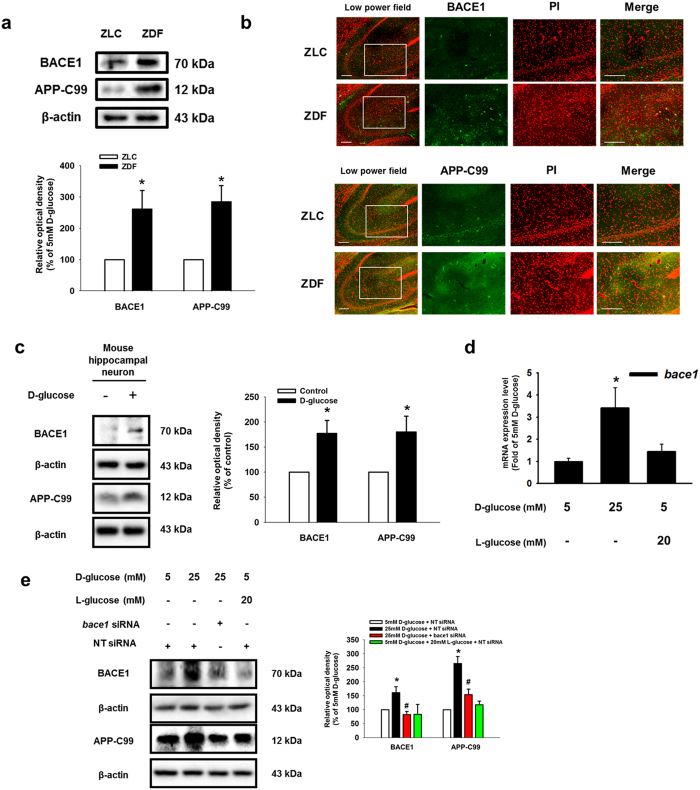
Effects of high glucose on BACE1 expression in diabetic rat models, mouse hippocampal neuron and SK-N-MC. (**a**) BACE1 and APP-C99 expressions of the ZLC and ZDF brain tissues were detected by western blotting. Each data shown in the result is representative images of five independent experiments. (**b**) Tissue samples for immunohistochemistry were immunostained with BACE1 and APP-C99 specific antibodies. Images shown in result are representative. All scale bars, 200 μm (magnification of low and high power field, ×100 and ×200). (**c**) Mouse hippocampal neuron was incubated with 25 mM D-glucose for 24 h BACE1, APP- C99 and β-actin expressions of mouse hippocampal neuron were detected by western blotting. Each data shown in the result is representative image of five independent experiments. (**d**) Total mRNA extracted from SK-N-MC was reverse-transcribed, and subsequently *bace1* and *β-actin* mRNA expressions were amplified by PCR. The mRNA expression of *bace1* was analyzed by quantitative real-time PCR. The mRNA expression level was normalized by *β-actin* mRNA expression level. Data are presented as a mean ± S.E. of three independent duplex dishes. (**e**) *bace1* and non-targeting (NT) siRNA was transfected to cell for 12 h prior to D-and L-glucose treatment for 24 h BACE1, APP-C99 and β-actin expressions were detected by western blotting. Each western blot image is representative of three independent experiments. Data are presented as a mean ± S.E. *p < 0.05 versus 5 mM of D-glucose treatment. All western blot data were cropped and acquired under same experimental conditions.

To determine the effect of high glucose on Aβ expression *in vivo*, we performed immunohistochemical staining of the hippocampal region (*Cornu Ammonis*; CA1-CA3) of brain tissues of ZLC control and ZDF diabetic rat models. Protein expressions of Aβ and phosphorylated tau were more evident in ZDF brain tissue than in ZLC brain tissue (Fig. 1a). Among the immunohistochemical results, the numbers of Aβ and phosphorylated tau-positive cells were greater in ZDF brain tissue than in ZLC brain tissue (Fig. 1b). To examine the effect of high glucose *in vitro*, SK-N-MC cells were treated with various concentrations of D-glucose (5–25 mM) for 24 h. By undertaking immunoprecipitation of Aβ in the medium, we observed that the high glucose dose (D-glucose; 25 mM) stimulated Aβ secretion. However, 20 mM of L-glucose treatment did not affect Aβ secretion (Fig. 1c). As shown in Fig. 1d, the fluorescence intensity of p-tau at the serine 396 residue increased to 206% by 25 mM D-glucose, not 25 mM L-glucose treatment. In addition, we checked tau phosphorylation and associated kinases to determine the effect of increased Aβ secretion resulting from high glucose treatment. Our results showed that D-glucose treatment induced phosphorylation of GSK-3β and AKT, a major kinase involving in tau phosphorylation^30^,^31^,^32^,^33^,^34^ (Sup. Fig. 4a,b). Furthermore, we examined the effect of D-glucose on apoptosis of SK-N-MC cells. The trypan blue exclusion assay results showed that D-glucose, not L-glucose, decreased SK-N-MC cell viability in a dose-dependent manner (Fig. 1e). Consistent with that result, our annexin V/PI FACS analysis showed that high glucose increased annexin V-positive or PI-positive SK-N-MC cells in a dose-dependent manner (Fig. 1f). Next, we assessed the expressions of BACE1, Presenilin-1 (PSEN1) and APP-C99 to investigate the effect of high glucose on Aβ secretion on the regulation of enzyme expression *in vivo* and *in vitro*. Our western blot analysis results showed that BACE1 and APP-C99 expressions were greater in ZDF rats than in ZLC rats (Fig. 2a). The immunohistochemical results showed the number of BACE1 and APP-C99 positive cells to be significantly higher in ZDF than in ZLC rat models (Fig. 2b). However, there were no significant differences in PSEN1 expression levels or PSEN1-positive cell numbers in the brain tissues of ZLC and ZDF rat models (Sup. Fig. 5a,b). In our results with mouse hippocampal neuron, BACE1 and APP-C99 expressions were induced by D-glucose treatment (Fig. 2c). Consistent with the *in vivo* results, high glucose increased the *bace1* mRNA expression level (Fig. 2d). And, both BACE1 and APP-C99 expressions were increased by D-glucose, but L-glucose treatment (Fig. 2e). In addition, D-glucose induced phosphorylation of AKT and GSK-3β, dephosphorylated by BACE1 siRNA transfection (Sup. Fig. 4b). AKT inactivation by wortmannin pretreatment reduced GSK-3β and tau phosphorylation (Sup. Fig. 4c). Furthermore, we found that we treated D-glucose to various cell lines including SK-N-MC, MEF and CACO-2. In our result, 25 mM D-glucose treatment increased BACE1 expression in SK-N-MC, but is ineffective in MEF and CACO-2 (Sup. Fig. 6). On the basis of our vivo and vitro data, we suggest that a high glucose level stimulates BACE1 expression and activation, which then leads to Aβ production in neuronal cell.

### Involvement of high glucose-induced HIF-1α expression in BACE1 expression

**Figure 3:**
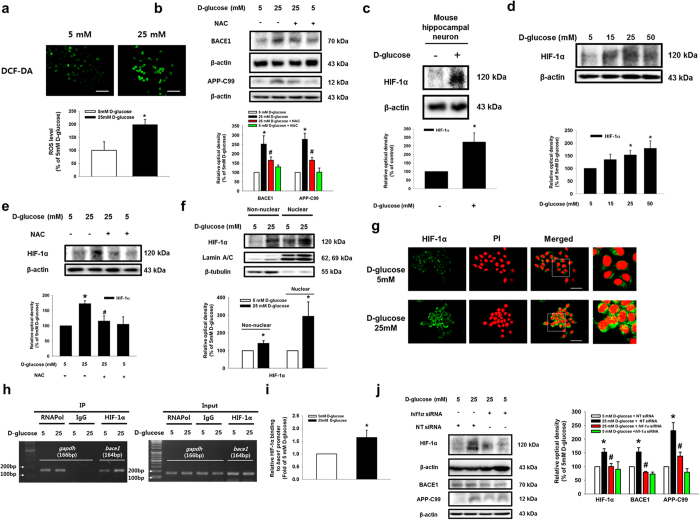
Role of ROS-induced HIF-1α in BACE1 expression by high glucose. (**a**) DCF-DA–sensitive SK-N-MC cells were visualized by confocal microscopy. Scale bars, 50 μm (magnification, ×600). Intracellular ROS generation was quantified by using luminometer. Data are presented as a mean ± S.E. of two independent sixth dishes. (**b**) BACE1, APP-C99 and β-actin expressions were detected by western blotting. (**c**) Mouse hippocampal neuron was incubated with 25 mM D-glucose for 24 h HIF-1α and β-actin expressions of mouse hippocampal neuron were detected by western blotting. Each data shown in the result is representative image of five independent experiments. (**d,e**) NAC (5 mM) was pretreated to cells, and then HIF-1α and β-actin expressions of SK-N-MC were detected by western blotting. (**f**) Non-nuclear protein and nuclear expressions were normalized by β-tubulin and lamin A/C repectively. (**g**) Cells were immunostained with HIF-1α and PI. Scale bars, 50 μm (magnification, ×800). (**h**) DNA was immunoprecipitated with RNA polymerase, IgG and HIF-1α antibodies. The immunoprecipitation and input samples were amplified with primers of *gapdh* and *bace1* gene promoters. (**i**) Quantatative data was analyzed by real time PCR of two independent experiments of triple dishes. (**j**) SK-N-MC cells were transfected by siRNAs for 24 h prior to D-glucose treatment. HIF-1α, APP-C99, BACE1 and β-actin were detected by western blotting. Each western blot image shown is representative of three independent experiments. Data are presented as a mean ± S.E. *p < 0.05 versus 5 mM of D-glucose treatment, ^#^*p* < 0.05 versus 25 mM of D-glucose treatment. All western blot data were cropped and acquired under same experimental conditions.

Additionally, we examined the role of intracellular ROS generation in BACE1 expression of SK-N-MC cells. The DCF-DA staining assay results showed the intracellular ROS level was 198% of the 5 mM D-glucose level in the 25 mM high glucose treatment group (Fig. 3a). Moreover, ROS scavenger NAC pretreatment diminished the 25 mM D-glucose-induced BACE1 and APP-C99 protein expressions (Fig. 3b). As shown in Fig. 3c,d, high glucose treatment increased HIF-1α expression in mouse hippocampal neuron and SK-N-MC, whereas up-regulation of HIF-1α expression was reduced by NAC pretreatment (Fig. 3e). In addition, high glucose treatment stimulated nuclear translocation of HIF-1α (Fig. 3f,g). Our CHIP results showed that the high glucose treatment stimulated binding of HIF-1α to the hypoxia responsive element (HRE) of the *bace1* gene promoter, while silencing of HIF-1α protein expression inhibited high glucose-induced BACE1 expression (Fig. 3h–j). These results indicate that high glucose directly regulates BACE1 expression via HIF-1α.

### Role of high glucose-mediated ROS generation in LXR-mediated ABCA1 expression and cholesterol accumulation

**Figure 4:**
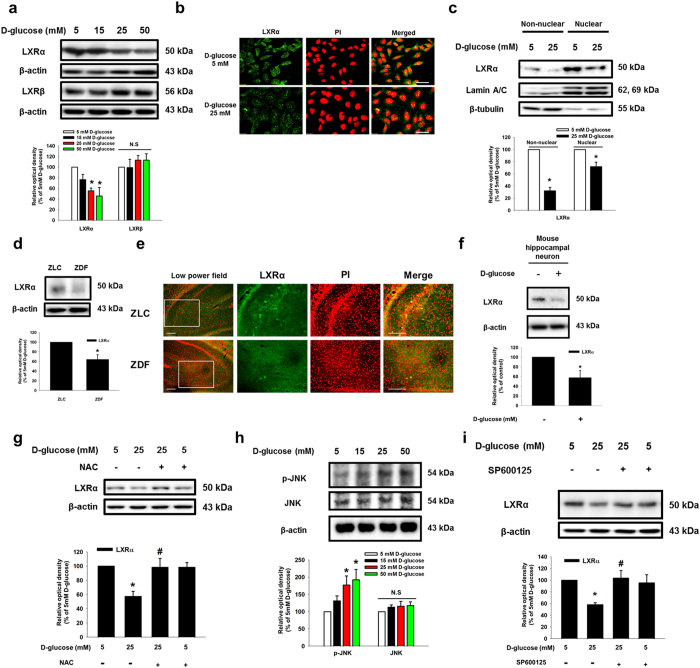
Role of high glucose-induced ROS in LXR reduction. (**a**) LXRα, LXRβ and β-actin were detected by western blotting. (**b**) Cells were immunostained with LXRα and PI. Scale bars, 50 μm (magnification, ×800). (**c**) LXRα, lamin a/c and β-tubulin in the non-nuclear and nuclear fractions were detected by western blotting. Non-nuclear protein and nuclear expressions were normalized by β-tubulin and lamin A/C repectively. (**d**) LXRα and β-actin protein expressions of the ZLC and ZDF brain tissues were detected by western blotting. (**e**) Immunohistochemistry with ZLC and ZDF tissues were performed with LXRα-specific antibody and PI. All scale bars, 200 μm (magnification of low and high power field, ×100 and ×200). (**f**) Mouse hippocampal neuron was incubated with 25 mM D-glucose for 24 h LXRα and β-actin expressions of mouse hippocampal neuron were detected by western blotting. Each data shown in the result is representative image of five independent experiments. (**g**) SK-N-MC was incubated with NAC (5 mM) for 30 min before D-glucose treatment. LXRα and β-actin expressions were analyzed by western blotting. (**h**) Phosphorylated JNK, JNK and β-actin expression were detected by western blotting. (**i**) SK-N-MC was incubated with SP600125 (1 μM) for 30 min before D-glucose treatment. LXRα expression was detected by western blotting. Each western blot image is representative of three independent experiments. Data are presented as a mean ± S.E. *p < 0.05 versus 5 mM of D-glucose treatment, ^#^*p* < 0.05 versus 25 mM of D-glucose treatment. All western blot data were cropped and acquired under same experimental conditions.

**Figure 5:**
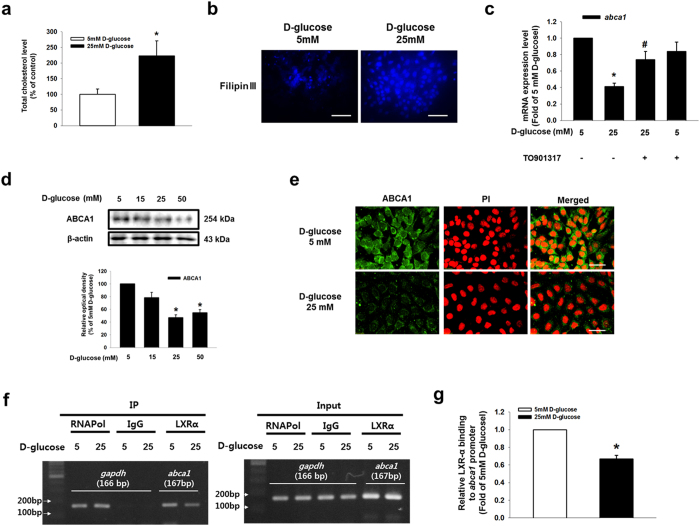
Role of high glucose-reduced LXRα in ABCA1 expression and cholesterol accumulation. (**a**) Total cholesterol level is measured by using cholesterol detection kit described in Materials & Methods. Data are presented as a mean ± S.E. of three independent experiments of duplex dishes. (**b**) Intracellular cholesterol was stained with fillipinIII, and visulalized by confocal microscopy. Scale bars, 50 μm (magnification, ×800). (**c**) TO901317 (1 μM) pretreated to SK-N-MC prior to D-glucose treatment. Total mRNA expressions of *abca11* and *β-actin* were analyzed by quantitative real-time PCR. The mRNA expression level was normalized by *β-actin* mRNA expression level. Data are presented as a mean ± S.E. of three independent duplex dishes. (**d**) ABCA1 and β-actin expressions were detected by western blotting. (**e**) Cells were immunostained with ABCA1 and PI. Scale bars, 50 μm (magnification, ×800). (**f**) DNA was immunoprecipitated with RNA polymerase, IgG and LXRα antibodies. The immunoprecipitation and input samples were amplified with primers of *gapdh* and *abca1* gene promoters. (**g**) CHIP data was quantified by real time PCR of two independent experiments of triple dishes. Each western blot image is representative of three independent experiments. Data are presented as a mean ± S.E. *p < 0.05 versus 5 mM of D-glucose treatment, ^#^*p* < 0.05 versus 25 mM of D-glucose treatment. All western blot data were cropped and acquired under same experimental conditions.

To examine the effect of D-glucose on LXR in SK-N-MC cells, we determined the effect of high glucose on mRNA expressions of *lxrα* and *lxrβ* in SK-N-MC cells. Both *lxrα* and *lxrβ* mRNAs were expressed in SK-N-MC cells; however, their mRNA expression levels were not changed by high glucose treatment (Sup. Fig. 7a,b). We treated with various concentrations of D-glucose (5–50 mM) for 24 h and examined subsequent LXRα and LXRβ protein expressions. High glucose decreased LXRα expression in a dose-dependent manner, but LXRβ expression was not affected by the D-glucose treatment (Fig. 4a). Moreover, high glucose reduced nuclear translocation as well as fluorescence intensity of LXRα (Fig. 4b,c). In addition, based on western blot and immunocytochemistry analyses D-glucose increased RXRα expression in a dose-dependent manner (Sup. Fig. 7c,d). Western blot results from ZLC and ZDF rats showed that LXRα protein expression was lower in ZDF rats than in ZLC rats (Fig. 4d). Immunohistochemical results showed that the number of LXRα-positive cells was lower in ZDF rats than in ZLC rats, but the number of RXR-positive cells was higher in ZDF rats than in ZLC rats (Fig. 4e and Sup. Fig. 7e). Consistent with these results, LXRα expression in mouse hippocampal neuron was reduced by high glucose treatment (Fig. 4f). As shown in Fig. 4g, a decrease in LXRα expression by high glucose treatment was reversed by pretreatment with ROS scavenger NAC. In addition, D-glucose stimulated JNK phosphorylation at the Thr^183^ and Tyr^185^ residues in a dose-dependent manner (Fig. 4h). High glucose-reduced LXRα expression was reversed by pretreatment with JNK inhibitor SP600125, but not with ERK inhibitor PD98059 (Fig. 4i and Sup. Fig. 8a). Furthermore, we performed immunoprecipitation to investigate the effect of high glucose on LXRα/RXRα heterodimer formation, and the results indicated that high glucose disrupted LXRα/RXRα heterodimer formation (Sup Fig. 8b). Those results suggest that high glucose-induced ROS generation contributes to a decrease in LXRα expression through the JNK pathway, and is followed by inhibition of LXRα nuclear translocation and LXRα/RXRα heterodimer formation. Next, we investigated the role of LXRα in intracellular cholesterol regulation. Our results from cholesterol measurement assaying and filipin III staining showed the intracellular cholesterol level in 25 mM D-glucose-treated SK-N-MC cells increased to 223% of that in 5 mM D-glucose-treated cells (Fig. 5a,b). The high glucose treatment reduced *abca1* mRNA expression, but not the *abcg1* mRNA expression; moreover, the *abca1* mRNA expression was reversed by LXR agonist TO901317 pretreatment (Fig. 5c and Sup. Fig. 9). In addition, D-glucose decreased ABCA1 protein expression in a dose-dependent manner (Fig. 5d). Furthermore, the fluorescence intensity of ABCA1 was decreased by the high glucose treatment (Fig. 5e). By using the CHIP assay, we confirmed high glucose-induced binding of LXR to the LXR element (LXE) region of the *abca1* promoter was blocked by TO901317 pretreatment (Fig. 5f,g, and Sup. Fig. 11).

### Role of localization of BACE1 on the lipid raft in amyloidogenesis and apoptosis of SK-N-MC

**Figure 6:**
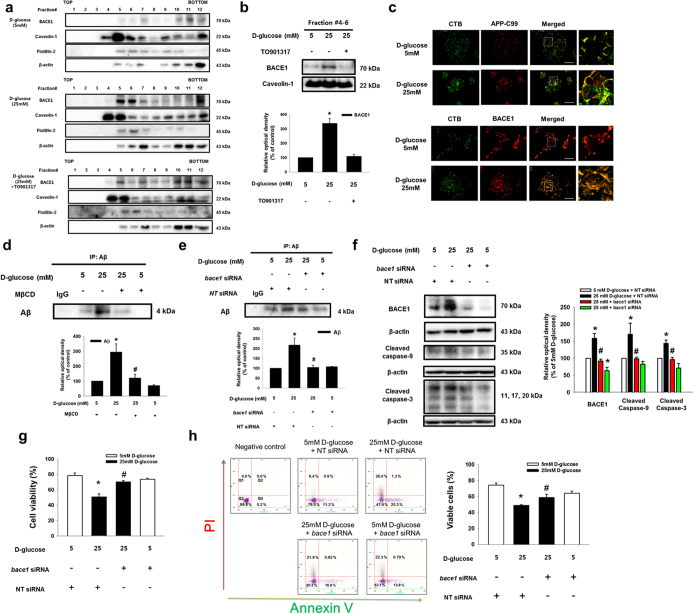
Role of high glucose-induced BACE1 localization on the lipid raft in amyloidogenesis and apoptosis of SK-N-MC. (**a**) SK-N-MC was incubated with TO901317 (1 μM) for 30 min prior to D-glucose treatment. Sucrose gradient-fractionized samples were blotted with BACE1, caveolin-1, flotillin-2 and β-actin-specific antibodies. (**b**) The same volumes of lipid raft fraction (#4–6) were loaded to SDS-PAGE gel, blotted with BACE1 and caveolin-1-specific antibodies. (**c**) Cells were stained with APP-C99, BACE1 and CTB, visualized by confocal microscopy. Scale bars, 50 μm (magnification, ×800). (**d,e**) Cells were incubated with MβCD and transfected with *bace1* and NT siRNAs prior to D-glucose treatment. Secreted Aβ in medium was analyzed by immunoprecipitation assay. (**f**) The *bace1* and NT siRNAs-transfected SK-N-MC samples were blotted with BACE1, cleaved caspase-9, cleaved caspase-3 and β-actin-specific antibodies. (**g**) Cell viability was measured by trypan blue exclusion assay. Data are presented as a mean ± S.E. of three independent experiments with duplex dishes. (**h**) Viable cells were detected by using annexin V/PI analysis. Data are presented as a mean ± S.E. of two independent duplex dishes. Each western blot image is representative of three independent experiments. *p < 0.05 versus 5 mM of D-glucose treatment, ^#^*p* < 0.05 versus 25 mM of D-glucose treatment. All western blot data were cropped and acquired under same experimental conditions.

To clarify the effect of high glucose on BACE1 localization, we performed sucrose gradient fractionation and immunocytochemical staining. As shown in Fig. 6a, high glucose stimulated translocation of BACE1 from the non-lipid raft part (fractions #7–#12) to the lipid raft part (fractions #4–#6). BACE1 expression in the lipid raft part (fractions #4-#6) was significantly increased by high glucose treatment (Fig. 6b). In addition, high glucose stimulated BACE1 and APP-C99 localizations in the lipid raft after being disrupted by TO901317 pretreatment (Fig. 6c). In addition, lipid raft disruption by MβCD pretreatment inhibited Aβ secretion (Fig. 6d). To confirm the effect of high glucose-induced BACE1 expression on amyloidogenesis and cell viability, we transfected *bace1* siRNA into SK-N-MC cells. As shown in Fig. 6e, high glucose-induced Aβ secretion was reduced by knockdown of BACE1 expression. In addition, we determined the expressions of apoptosis markers caspase-9 and 3 to examine the role of high glucose-induced BACE1 expression in apoptosis of SK-N-MC cells. Our results showed that silencing of BACE1 expression inhibited expressions of cleaved caspase-9 and 3 (Fig. 6f). We confirmed that high glucose-induced apoptosis of SK-N-MC cells was decreased by inhibition of BACE1 expression by performing trypan blue exclusion assays and annexin V/PI FACS analyses (Fig. 6g,h). Based on these results, our findings indicate that high glucose-induced BACE1 localization and expression on the lipid raft is important for Aβ secretion and apoptosis of SK-N-MC cells.

## Discussion

**Figure 7:**
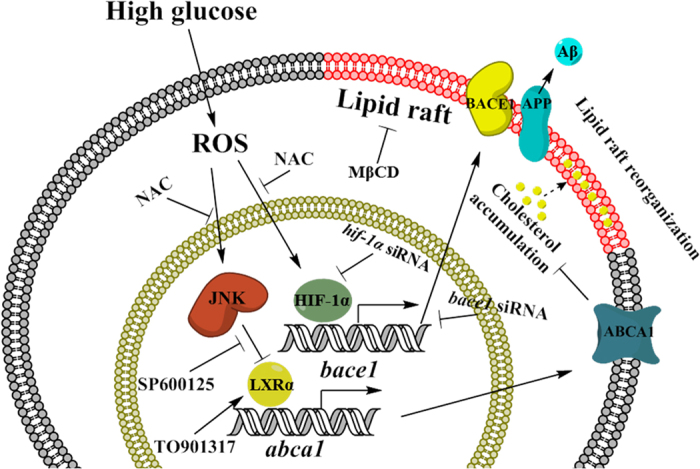
The schematic model for mechanism involved in high glucose-induced BACE1 expression and amyloidogenesis of SK-N-MC. High glucose stimulates ROS production, leads to increase of HIF-1α for nuclear translocation and phosphorylation of JNK. Translocated HIF-1α is bound to HRE promoter region of *bace1* gene, subsequently increases BACE1 expression. Phosphorylated JNK stimulates decrease in LXRα expression, reduced binding LXRα to the LXE promoter region of *abca1* gene, leads to reduction of ABCA1 expression and intracellular cholesterol accumulation which may be involved in high glucose-induced lipid raft reorganization. In addition, high glucose-lipid raft modification induces BACE1 expression on the lipid raft, stimulates to Aβ secretion in SK-N-MC.

In the present study, we have shown that high glucose-induced BACE1 expression and the reorganization of the lipid raft through cholesterol accumulation stimulate Aβ production (Fig. 7). Our results show that the brain of ZDF rats exposed to high glucose exhibits increases in Aβ deposition and tau phosphorylation levels, which suggest the existence of a connection between two main pathophysiological features of AD; that is, Aβ production/secretion and tau phosphorylation. Thus, we investigated how a high glucose level affects APP processing in neuronal cell with mouse hippocampal neuron and SK-N-MC. Consistent with the ZDF rat model results, high glucose (D-glucose; 25 mM), but not high L-glucose (20 mM), stimulated Aβ production and tau phosphorylation, which suggests that the *in vitro* experimental model using SK-N-MC cells reflects an *in vivo* diabetic condition. A low D-glucose level (5 mM) was used as a control, and reflects the normal plasma glucose concentration of non-diabetic individuals. According to previous study, fasting blood glucose level in lean subjects maintains a narrow range of approximately 4.8 ± 0.1 mmol/L, whereas type 2 diabetes patients present a high fasting plasma glucose level of 11.1 ± 0.9 mmol/L^35^. In addition, our results showed that high glucose-induced Aβ production, tau phosphorylation, and apoptosis are mainly dependent on alteration of glucose metabolism rather than on an osmotic effect. Consistent with our findings, previous investigators have reported that the hyperglycemic condition in DM is closely linked to diabetic neuropathy and apoptosis^36^,^37^. Moreover, we presented that high glucose induced SK-N-MC apoptosis in a dose dependent manner. In the previous reports, it is suggested that high glucose treatment or hyperglycemic condition induces ROS and BACE1-mediated Aβ production, critical to high glucose-induced neuronal cell apoptosis^38^,^39^,^40^,^41^.

In this study, we found that ZDF rats exhibited higher levels of APP-C99 and BACE1 expressions than those in ZLC rats, and that high glucose treatment stimulated APP-C99 and BACE1 expressions in mouse hippocampal neuron and SK-N-MC. These results indicate that a high glucose level itself is a risk factor for AD through the regulation of BACE1. As shown in Sup. Fig. 4a–c, our observation suggest the possibility that high glucose-induced BACE1 expression regulates AKT phosphorylation, involved in GSK-3β phosphorylation at Ser^9^ and tau phosphorylation at Ser^396^. It has been reported that phosphorylation of AKT and GSK3β might be associated with tau phosphorylation at Ser^396^, and the GSK-3β phosphorylation at Ser^9^ is a downstream target of AKT activation^42^,^43^,^44^. Although the phosphorylation of GSK-3β at Ser^9^ has an inhibitory role in GSK3β activity, there have been several reports suggesting induction of oxidative stress and inactivation of PP2A increase GSK-3β phsphorylation at Ser^9^ and activity via another alterative pathway such as a calpain activation, which is important for tau phosphorylation in AD brain^31^,^45^,^46^,^47^,^48^,^49^,^50^. In addition, our results indicate that high glucose-induced BACE1 expression is mainly regulated by ROS generation and HIF-1α expression. Oxidative stress, resulting from hyperglycemia-induced ROS generation under diabetic conditions, has been associated with various neurodegenerative diseases including AD^51^. Thus, deciphering the underlying molecular mechanisms involved in the effect of high glucose-induced oxidative stress on AD pathogenesis is necessary for elucidating the linkage between DM and AD. It has been reported that the alteration of glucose metabolism is tied to the activation of the transcription factor HIF-1α, mediated by an increase in ROS generation^51^,^52^. Interestingly, both a previous report and our data presented in Sup. Fig. 10 suggest that a functional HRE is present in the *bace1* promoter^53^. In addition, another investigation reported that upregulated HIF-1α increased mRNA and protein expression of BACE1, but that *hif-1α* knock-out mouse exhibit a decrease in BACE1 expression in the hippocampus, suggesting a link between HIF-1α and Aβ processing under diabetic conditions^54^. Consistent with those results, our data showed that ZDF rats exhibit a high level of BACE1 expression, and that a high glucose condition stimulates the binding of HIF-1α to the *bace1* gene promoter, and subsequently increases the *bace1* promoter transcription level. These findings suggest that a high glucose condition stimulates Aβ production through HIF-1α-dependent direct expression of BACE1, and high glucose-induced oxidative stress has a critical role in the pathogenesis of AD.

Based on our study results, we suggest that high glucose decreases ABCA1 expression through JNK-reduced LXRα expression and binding to the *abca1* gene promoter. Although the role of ROS in LXR expression or activation has not been completely described, several researchers have reported that JNK, which can be activated by ROS, regulates LXR activation^55^,^56^. In addition, LXR phosphorylation by JNK stimulates the export of liver rich transcription factors, including LXR, which has a nuclear export signal-containing DNA-binding domain from the nucleus and inactivates LXR for degradation in the cytoplasm^57^,^58^,^59^,^60^. These findings suggest the possibility that ROS-induced JNK phosphorylation resulting from high glucose levels stimulates export of inactivated LXR from the nucleus, leading to degradation, and is responsible for high glucose-reduced LXRα expression. Moreover, we found that high glucose reduced LXRα only, not LXRβ, even though high glucose did not affect mRNA expressions of either LXRα or LXRβ. A previous investigation demonstrated that LXRα and LXRβ have similar protein structures, but their nuclear localization is differently controlled^61^, suggesting that differently regulated nuclear localization of LXRs could contribute to high glucose-reduced LXRα expression. Furthermore, our results showed that formation of a LXRα/RXRα heterodimer and binding of LXRα to LXE of the *abca1* promoter were reduced by high glucose, even though high glucose increased RXRα expression. It has been well documented that activated LXR stimulates formation of a heterodimer with RXR and controls transcriptional activity of the *abca1* gene^62^,^63^. Indeed, Repa *et al*. reported pharmacological activation of LXR with an LXR agonist can induce ABCA1 expression via LXR/RXR heterodimer formation^64^. These findings indicate that high glucose-inhibited formation of the LXRα/RXRα heterodimer can control ABCA1 expression. However, additional investigation into the role of increased RXRα by high glucose in AD pathogenesis is needed.

The present study has shown that high glucose can increase the intracellular cholesterol level, and that action is associated with LXR/ABCA1-mediated lipid raft reorganization. Although there are reports that reverse cholesterol transport (RCT) by LXR is involved in the pathological condition in AD, with the exception of atherosclerosis in mice, the role of RCT in AD pathogenesis under diabetic condition has not been fully described^65^. Regardless, previous reports and the results of the present study suggest that high glucose-induced LXR-mediated cholesterol accumulation is closely related to progression in AD. In addition, our results showing that high glucose increases expression of BACE1, located in the lipid raft, suggests the possible role of cholesterol and lipid raft on APP processing by BACE1. As cholesterol serves as a structural part between sphingolipid hydrocarbon chains in the lipid raft, alteration of the cholesterol content of cells can result in lipid raft reorganization. In fact, several previous investigations have demonstrated that altered free cholesterol and plasma membrane cholesterol have an influence on lipid raft structure and functions^66^,^67^,^68^, suggesting that the alteration of intracellular cholesterol level could be an important regulator of lipid raft reorganization. In the present study, high glucose stimulated changes in cholesterol in the lipid bilayer and affected APP processing through regulation of lipid raft reorganization, which elicits BACE1 activity and thus stimulates Aβ production and apoptosis of SK-N-MC cells. Consistent with these results, there is mounting evidence that cholesterol has a critical role in the regulation of BACE1 activity and the cleavage of APP. In addition, it has been reported that an increase in dietary cholesterol uptake stimulates amyloid plaque formation, while the 3-hydroxy-3-methylglutaryl-CoA–reductive (HMG-CoA–reductase) inhibitor relieves the neurological symptoms of AD^69^. These previous and current findings suggest that regulation of high glucose-induced lipid raft reorganization plays an important role in Aβ production via regulation of BACE1 activity, and such regulation has potential as a novel therapeutic approach to controlling BACE1 expression and activation thereby leading to neuronal cell death.

Taken together, our observations with ZLC/ZDF rat brain, mouse hippocampal neuron and SK-N-MC cell line suggest the possible role of high glucose in Aβ production as a risk factor associated with AD occurrence in diabetic patients. Furthermore, the high glucose-induced increase in HIF-1α and decrease in LXRα-mediated cholesterol accumulation appear to act as intermediate regulators linking DM and AD. Therefore, those affected pathways could be used in the development of therapeutic strategies and identification of selective targets for prevention of AD occurrence in DM patients. In conclusion, high glucose stimulates BACE1 through HIF-1α expression and LXRα/ABCA1-mediated localization in the lipid raft, actions that are important for high glucose-induced Aβ production and neuronal cell apoptosis.

## Additional Information

**How to cite this article**: Lee, H. J. *et al*. High glucose upregulates BACE1-mediated Aβ production through ROS-dependent HIF-1α and LXRα/ABCA1-regulated lipid raft reorganization in SK-N-MC cells. *Sci. Rep*. **6**, 36746; doi: 10.1038/srep36746 (2016).

**Publisher’s note**: Springer Nature remains neutral with regard to jurisdictional claims in published maps and institutional affiliations.

## Supplementary Material

Supplementary Information
