# Supplementary material for: High glucose upregulates BACE1-mediated Aβ production through ROS-dependent HIF-1α and LXRα/ABCA1-regulated lipid raft reorganization in SK-N-MC cells
Source: Sci Rep. 2016 Nov 10;6:36746. doi: 10.1038/srep36746 (PMC5103190; doi:10.1038/srep36746)
Supplement: Supplementary Information [file srep36746-s1.pdf]

Title: High glucose upregulates BACE1-mediated Aβ production through ROS-dependent HIF-1α and LXRα/ABCA1-regulated lipid raft reorganization in SK-N-MC cells

Hyun Jik Lee[1], Jung Min Ryu[2], Young Hyun Jung[1], Sei-Jung Lee[1], Jeong Yeon Kim[1], Sang Hun Lee[3,4], In Koo Hwang[5,6], Je Kyung Seong[5,6] and Ho Jae Han[1,*]

Affiliation

[1]Department of Veterinary Physiology, College of Veterinary Medicine, Research Institute for Veterinary Science and BK21 Creative Veterinary Research Center, Seoul National University, Seoul 08826, Korea.

[2]Department of Veterinary Physiology, College of Veterinary Medicine, Chonnam National University, Gwangju 61186, Korea.

[3]Medical Science Research Institute, Soonchunhyang University Seoul Hospital, Seoul, Republic of Korea.

[4]Department of Biochemistry, Soonchunhyang University College of Medicine, Cheonan, 330-930, Republic of Korea.

[5]BK21 PLUS Program for Creative Veterinary Science Research, and Research Institute for Veterinary Science; Seoul National University; Seoul, Korea.

[6]Department of Anatomy and Cell Biology; Korea Mouse Phenotyping Center (KMPC); College of Veterinary Medicine; Seoul National University; Seoul, Korea.

[*]Corresponing author: Ho Jae Han, D.V.M., Ph.D.

Department of Veterinary Physiology, College of Veterinary Medicine and Research Institute for Veterinary Science, Seoul National University, Seoul 08826, Korea

Tel: +82-02-880-1261

Fax: +82-2-880-2732

E-mail: hjhan@snu.ac.kr

Author contributions

Lee.HJ: Conception and design, Collection and/or assembly of data, Data analysis and interpretation, Manuscript writing

Ryu.JM: Conception and design, Collection and/or assembly of data, Data analysis and interpretation, Manuscript writing

Jung.YH: Collection and/or assembly of data, Manusript writing

Lee.S: Conception and design, Manuscript writing

Kim.JY: Manuscript writing

Lee.SH: Manuscript writing

Hwang.IK: Data analysis and interpretation

Sung.JK: Data analysis and interpretation

Han.HJ: Conception and design, Data analysis and interpretation, Manuscript writing

Supplemental figure legends

Supplemental figure 1. Full-length western blot images for key data in figures 1-3.

All western blot images are full-length blot imagesof key blot data in the figures 1-3. Dash line box indicates cropped blot image in the figures 1-3.

Supplemental figure 2. Full-length western blot images for key data in figures 4-6.

All western blot images are full-length blot images of key blot data in the figures 4-6. Dash line box indicates cropped blot imagein the figures 4-6.

Supplemental figure 3. Validation of specificity of Aβ antibody.

SK-N-MCs were treated with 10uM of L-685,458 for 24 h. And then, the medium samples were collected. Aβ secretion was analyzed by immunoprecipitation and western blotting. A blot result shown is representative images of three independent experiments.

Supplemental figure 4. Role of AKT and GSK-3β phosphorylation in BACE1-induced tau phosphorylation by high glucose.

(a) SK-N-MCs were treated with various concentrations (5-50 mM) of D-glucose for 24. And then, p-GSK3β (Ser^9), GSK3β and β-actin were detected by using western blotting. (b) SK-N-MC was incubated with bace1 specific siRNA prior to 25mM D-glucose treatment for 24

hr. p-AKT (Thr^{308} and Ser^{473}), AKT and β-actin were detected by using western blotting. Cells were incubated with wortmannin (10μM) prior to D-glucose treatment for 24 hr. p-GSK3β (Ser^{9}), GSK3β, p-Tau (Ser^{396}), Tau and β-actin were detected by using western blotting. Each blot result shown is representative images of three independent experiments. Data are presented as a mean ± S.E. *p< 0.05 versus 5 mM of D-glucose treatment, #p< 0.05 versus 25 mM of D-glucose treatment.

Supplemental figure 5. PSEN1 and BACE1 expression in vivo and in vitro.

(a) PSEN1 and β-actin protein expressions of the ZLC and ZDF brain tissues were detected by western blotting. Each blot result shown is representative images of five independent experiments. (b) Slide samples for immunohistochemistry were stained with PSEN1-specific antibody and PI. Images shown in result are representative. All scale bars, 200μm (magnification of low and high power field, ×100 and ×200). All western blot data were cropped and acquired under same experimental conditions.

Supplemental figure 6. Effect of high glucose on BACE1 expression in various cell lines

SK-N-MC, MEF, CACO-2 cell lines were incubated with 5 or 25 mM of D-glucose for 24 h. BACE1 and β-actin were detected by using western blotting. Each blot result shown is representative images of three independent experiments.

Supplemental figure 7. Effect of high glucose on *lxrα* and *lxrβ* mRNAs and RXRα expression.

(a) Extracted mRNA of SK-N-MC was reverse-transcribed, and amplified by PCR with primers of *lxrα*, *lxrβ* and *β-actin*. (b) SK-N-MC was incubated with D-glucose (5 and 25 mM). The *lxrα andlxrβ* mRNA expression levels were measured by real time PCR.Data are presented as a mean ± S.E of two independent experiments of triple dishes. (c) RXRα was detected by western blotting. (d) SK-N-MC was stained with RXRα-specific antibody and PI, visualized by confocal microscopy. Confocal images shown in result are representative. Scale bars, 50 μm (magnification, ×800). (e) Slide samples of rat brain tissues for immunohistochemistry were stained with RXRα-specific antibody and PI, visualized by confocal microscopy. All scale bars, 200μm (magnification of low power field and high power field, ×100 and ×200). Each western blot is representative of three independent experiments. Data are presented as a mean ± S.E. *p< 0.05 versus 5 mM of D-glucose treatment. N.S. Not statistically significant. All western blot data were cropped and acquired under same experimental conditions.

Supplemental figure 8. Role of ERK in high glucose-reduced LXRα expression and LXRα/RXRα heterodimer formation.

(a) SK-N-MC was treated with PD98059 (1μM) for 30 min prior to D-glucose (5 and 25 mM) treatment for 24 h. LXRα and β-actin were detected by western blotting. (b) SK-N-MC was treated with D-glucose (5 and 25 mM). Formation of LXRα/RXRα complex was detected by immunoprecipitation described in Materials & Methods section. Each western blot is representative of three independent experiments. Data are presented as a mean ± S.E. *p< 0.05 versus 5 mM of D-glucose treatment, #p< 0.05 versus 25 mM of D-glucose treatment. All western blot data were cropped and acquired under same experimental conditions.

Supplemental figure 9. Role of high glucose-reduced LXRα expression in *abcg1* mRNA expression.

SK-N-MC was incubated with TO901317 (1μM) for 30 min prior to D-glucose (5 and 25 mM) treatment. Extracted mRNA of SK-N-MC was reverse-transcribed, and amplified by PCR with primers of *abcg1* and *β-actin*. Expression levels of mRNA were analyzed by real time PCR. Data are presented as a mean ± S.E of two independent experiments of triple dishes. N.S. Not statistically significant.

Supplemental figure 10. The sites for LXRα binding to the LXE promoter region of the *abca1* gene.

The red colored sequence represents the consensus sequence of LXE.

Supplemental figure 11. The sites for HIF-1α binding to the HRE promoter region of the *bace1* gene.

The red-colored sequence represents the consensus sequence of HRE.

Supplemental figures

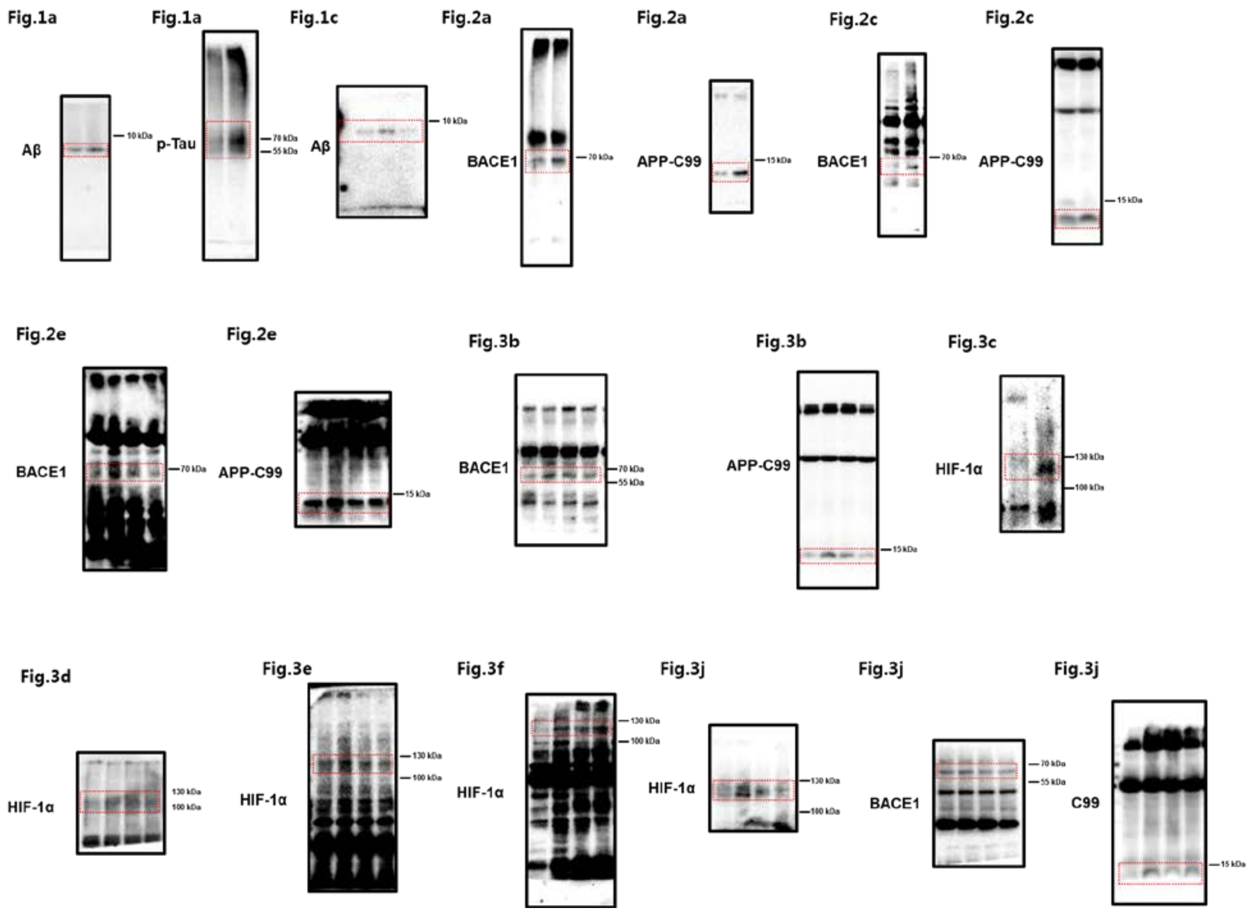

Supplemental figure 1.

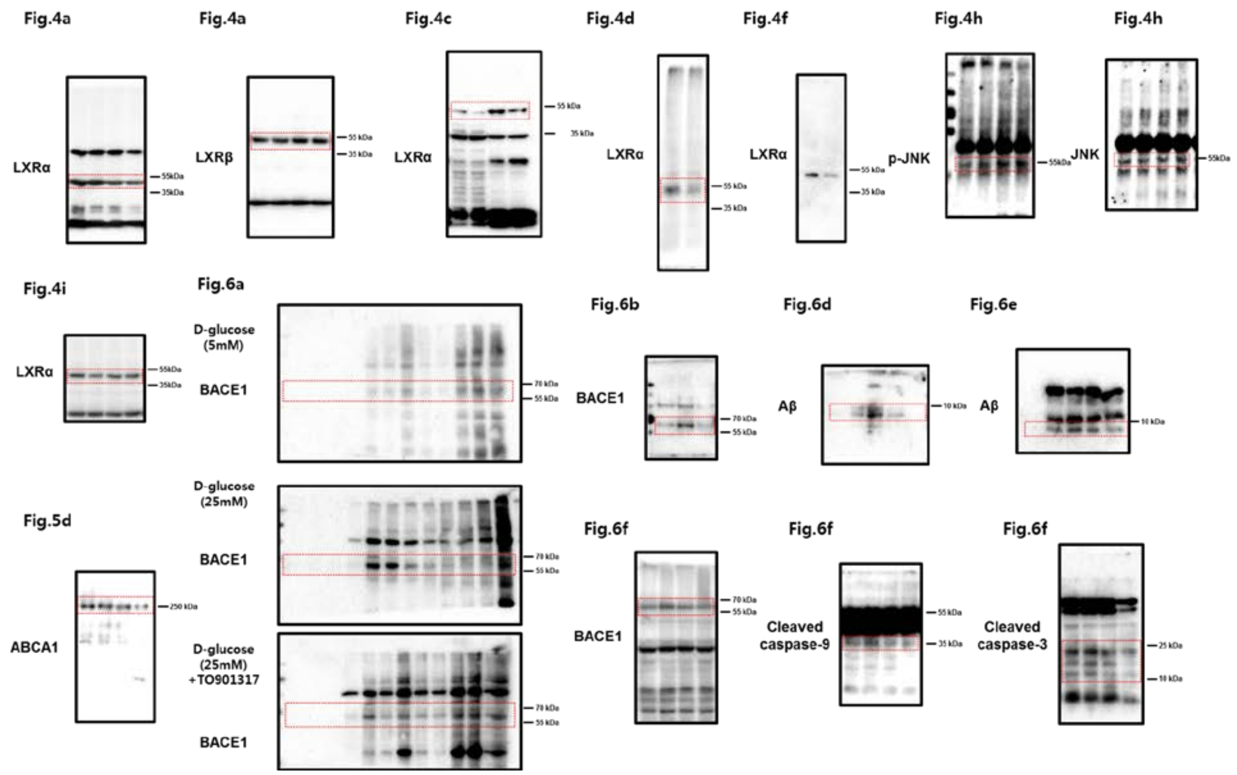

Supplemental figure 2.

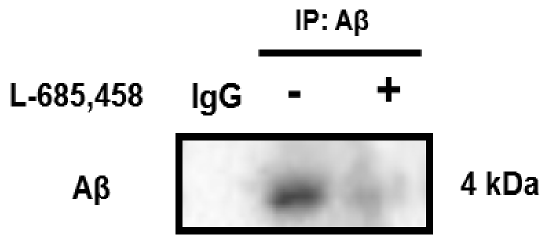

Supplemental figure 3.

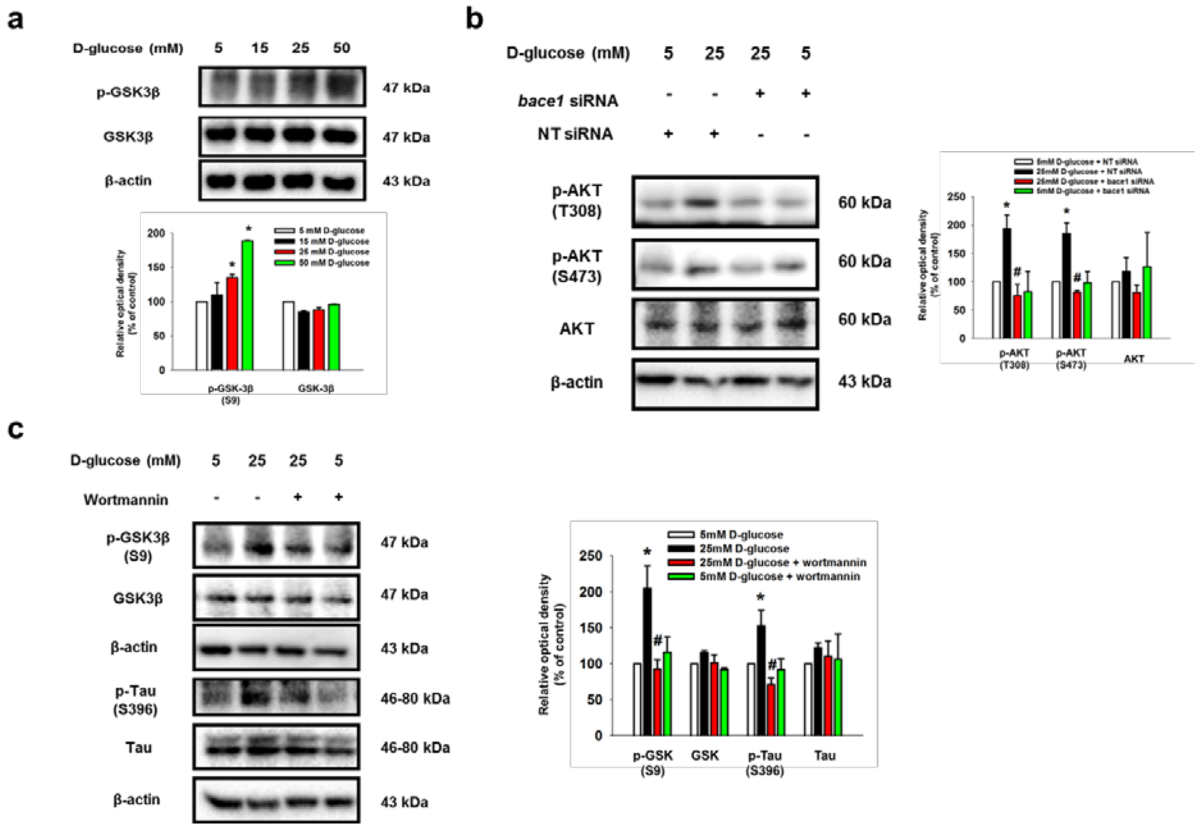

Supplemental figure 4.

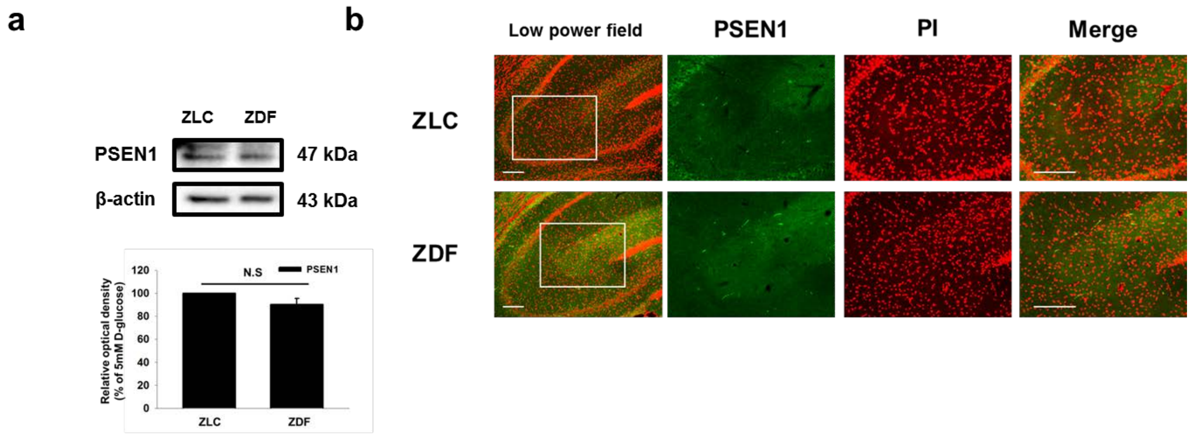

Supplemental figure 5.

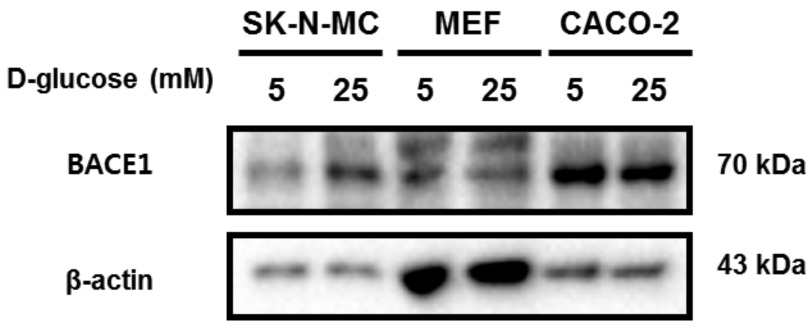

Supplemental figure 6.

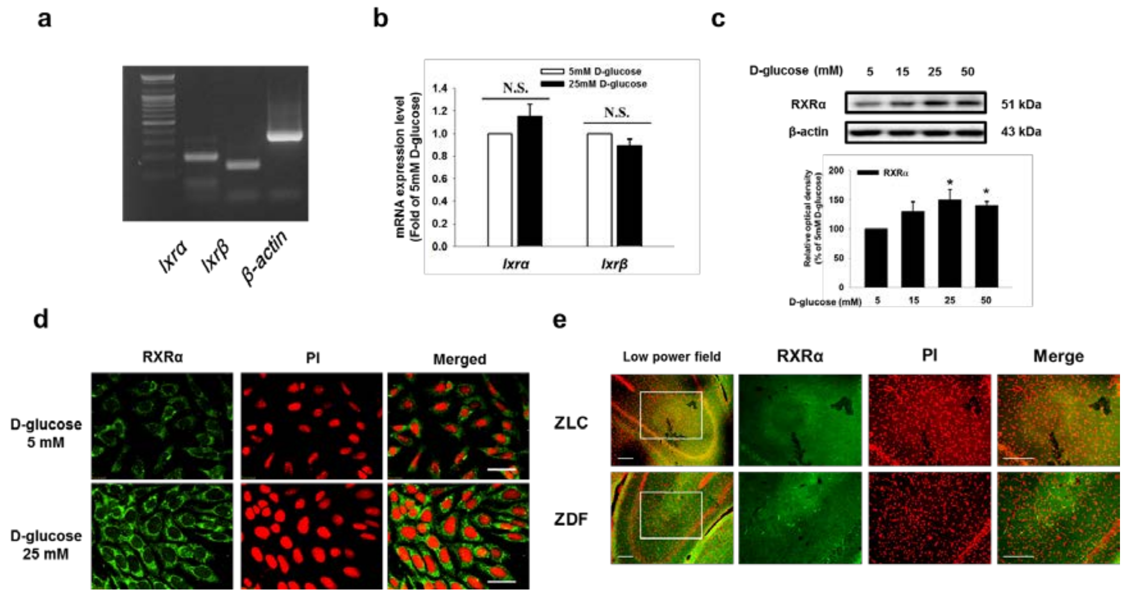

Supplemental figure 7.

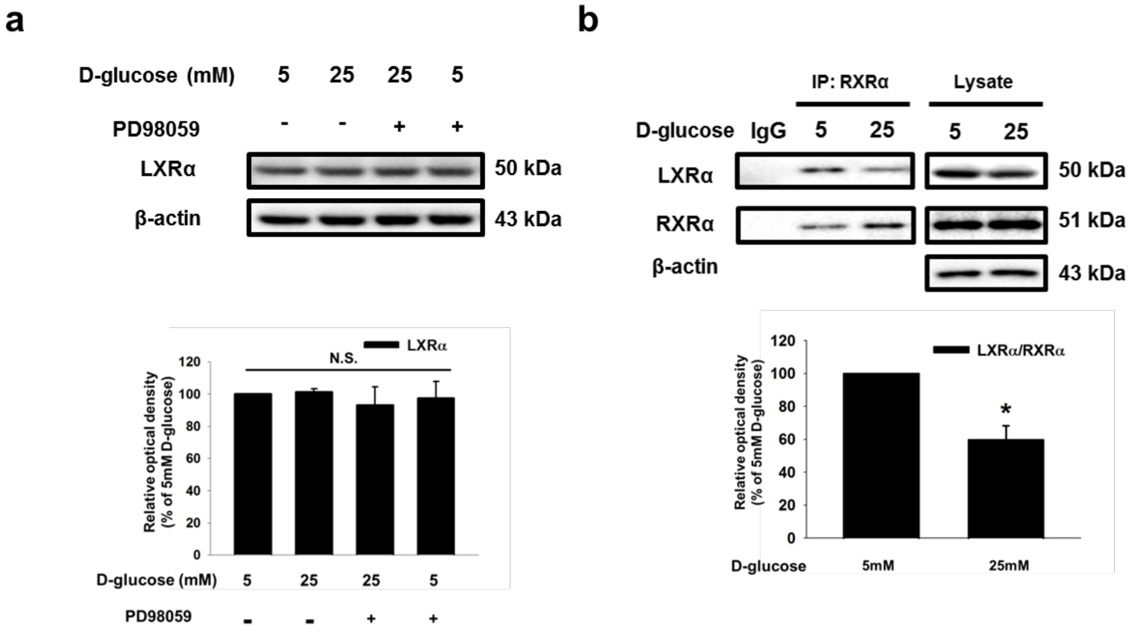

Supplemental figure 8.

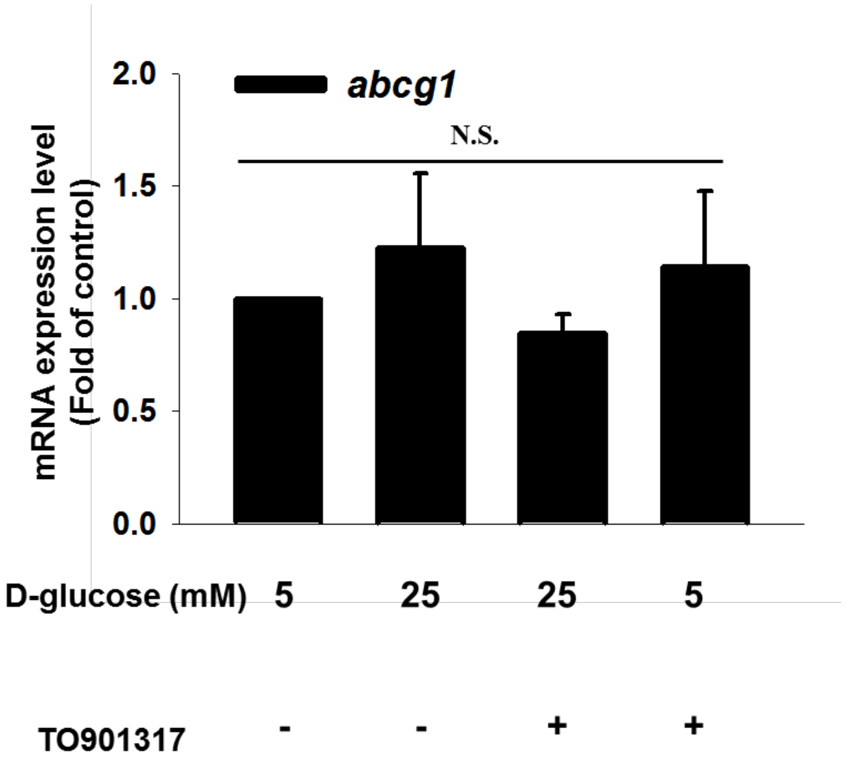

Supplemental figure 9.

```
-800 GAAATTAAAAGGGGGCTGGTCCATATTGTTCTGTGTTTTTGTTTGTTTGTTTCTTTTTTT
-740 GTTTTTGTGGCCTCCTTCCTCTCAATTTATGAAGAGAAGCAGTAAGATGTTCCTCTCGGG
-680 TCCTCTGAGGGACCTGGGGAGCTCAGGCTGGGAATCTCCAAGGCAGTAGGTCGCCTATCA
-620 AAAATCAAAGTCCAGGTTTGTGGGGGGAAAACAAAAGCAGCCCATTACCCAGAGGACTGT
-560 CCGCCTTCCCCTCACCCCAGCCTAGGCCTTTGAAAGGAAACAAAAGACAAGACAAAATGA
-500 TTGGCGTCCTGAGGGAGATTCAGCCTAGAGCTCTCTCTCCCCCAATCCCTCCCTCCGGCT
-440 GAGGAAACTAACAAAGGAAAAAAAAATTGCGGAAAGCAGGATTTAGAGGAAGCAAATTCC
-380 ACTGGTGCCCTTGGCTGCCGGGAACGTGGACTAGAGAGTCTGCGGCGCAGCCCCGAGCCC
-320 AGCGCTTCCCGCGCGTCTTAGGCCGGCGGGCCCGGGCGGGGGAAGGGGACGCAGACCGCG
-260 GACCCTAAGACACCTGCTGTACCCTCCACCCCCACCCCACCCACCTCCCCCCAACTCCCT
-200 AGATGTGTCGTGGGCGGCTGAACGTCGCCCGTTTAAGGGGCGGGCCCCGGCTCCACGTGC
-140 TTTCTGCTGAGTGACTGAACTACATAAACAGAGGCCGGGAACGGGGCGGGGAGGAGGGAG
-080 AGCACAGGCTTTGACCGATAGTAACCTCTGCGCTCGGTGCAGCCGAATCTATAAAAGGAA
-020 CTAGTCCCGGCAAAAACCCCG
```

Supplemental figure 10.

```
-500 TGCAGCCTGGAAAAACTCTTCCTCCCCTTTCATCCTGCTCCCTCAATCTCTGCTCGTGAA
-440 AATCCTACCCATTTTTAAACGCTCAAATTAAATACCACCTTCTCCCACTGAGTCTTCCCA
-380 GATCCCCTAGATGTCCCTCCAAATCCCTACTCCTTAATGCCACATCGTATAGTGTGAAAC
-320 CCCACCGCGGCAGGAATCACTTAACTGTCCCGTGTAGAATCACCGTTTATTTCTCTCTGT
-260 ATCTCCAGCGCCTGGCAGTGTGCCTGGCATACAGTGGGTGCTCCTTCCATGCTGAAAGAA
-200 AGACTGACAGACGGGAGGTGTGCCCCTCTCCATCCGTCTGGCCCTTCCCGCCAGGGCCTT
-140 GCAGGGCGGACTCCACCTCGGCAGAGGGCATCCCAGACCCCTCTCCAGCCCCGGAAGCCG
-080 GATTGCCTGCCATGGGAAGACTACACTTCCCAGCGATCCCAGGGAAAAGCGAAAACCTTT
-020 TGGCTTTGACAGCCGCCGCCA
```

Supplemental figure 11.

Supplemental Table 1. Sequences of primers used for RT-PCR and real-time PCR

Gene	Identification	Sequence (5'-3')	Size (bp)
bace1	Sense	CTGCCTGGATTTCTTCCTATTA	255
	Antisense	CTTGTGGTGGAGGACATAAG	
abca1	Sense	CGGTGCAGCCGAATCTATAA	286
	Antisense	CGCCGTGGCTGGTCATTA	
abcg1	Sense	TGCAATCTTGTGCCATATTTGA	118
	Antisense	CCAGCCGACTGTTCTGATCA	
β-actin	Sense	AACCGCGAGAAGATGACC	351
	Antisense	AGCAGCCGTGGCCATCTC	

Suppplemental Table 2. Sequences of siRNAs used for gene silencing

Target gene	Sequence 5'-3'
hif-1α	GCCGCUCAAUUUAUGAAUATT<br>UAUUCAUAAAUUGAGCGGCTT
	GCCUCUUUGACAAACUUAATT<br>UUAAGUUUGUCAAAGAGGCTT
	CCACCACUGAUGAAUUAAATT<br>UUUAAUUCAUCAGUGGUGGTT
	GCUGGAGACACAAUCAUAUTT<br>AUAUGAUUGUGUCUCCAGCTT
bace1	GCAAGGAGUACAACUAUGA
	GGAGGGAGCAUGAUCAUUG
	UAUGGGAGCUGUUAUCAUG
	AGACGACUGUUACAAGUUU
Non-targeting	UUCUCCGAACGUGUCACGUTT<br>ACGUGACACGUUCGGAGAATT

Supplemental Table 3. Sequences of CHIP primers used for RT-PCR and real-time PCR

Gene	Identification	Sequence (5'-3')	Size (bp)
*bace1*promoter	Sense	CCCTTTCATCCTGCTCCCTC	164
	Antisense	GCGGTGGGGTTTCACACTAT	
*abca1*promoter	Sense	GTTCCTCTCGGGTCCTCTGA	167
	Antisense	TTTCAAAGGCCTAGGCTGGG	
